# The cell biology of HIV-1 and other retroviruses

**DOI:** 10.1186/1742-4690-3-77

**Published:** 2006-11-03

**Authors:** Eric O Freed, Andrew J Mouland

**Affiliations:** 1 Virus-Cell Interaction Section, HIV Drug Resistance Program, National Cancer Institute, Frederick, MD. 21702-1201, USA; 2 HIV-1 RNA Trafficking Laboratory, Lady Davis Institute for Medical Research-Sir Mortimer B. Davis Jewish General Hospital, Departments of Medicine, Microbiology and Immunology, McGill University, Montréal, Québec, H3T 1E2, Canada

## Abstract

In recognition of the growing influence of cell biology in retrovirus research, we recently organized a Summer conference sponsored by the American Society for Cell Biology (ASCB) on the Cell Biology of HIV-1 and other Retroviruses (July 20–23, 2006, Emory University, Atlanta, Georgia). The meeting brought together a number of leading investigators interested in the interplay between cell biology and retrovirology with an emphasis on presentation of new and unpublished data. The conference was arranged from early to late events in the virus replication cycle, with sessions on viral fusion, entry, and transmission; post-entry restrictions to retroviral infection; nuclear import and integration; gene expression/regulation of retroviral Gag and genomic RNA; and assembly/release. In this review, we will attempt to touch briefly on some of the highlights of the conference, and will emphasize themes and trends that emerged at the meeting.

**Meeting report:**

The conference began with a keynote address from W. Sundquist on the biochemistry of HIV-1 budding. This presentation will be described in the section on Assembly and Release of Retroviruses.

## Viral fusion, entry, and transmission

**Eric Freed **opened the meeting by introducing work from his laboratory that identified the cholesterol-binding agent amphotericin B methyl ester (AME) as a potential compound to block HIV-1 replication [1]. Addition of AME to cultured cells inhibited HIV-1 replication in T cells and this group demonstrated that AME induced a block at the level of viral entry. However, extended viral kinetics revealed a recovery of HIV-1 replication. This was shown to be due to the emergence of AME-resistant mutants. Sequencing data revealed changes in the cytoplasmic tail of the transmembrane envelope glycoprotein, gp41. Truncation of gp41 also reversed the AME-imposed block to both HIV-1 and simian immunodeficiency virus (SIV) infection. Surprisingly, Eric Freed's group revealed that gp41 cleavage by the viral protease was responsible for the AME resistance.

**Walther Mothes **then introduced his work in mostly spectacular videomicroscopy clips and images on retrovirus transmission from an infected to an uninfected cell. He showed real-time video microscopy of murine leukemia virus (MLV) particles traveling or "surfing" on cytonemes that are long-lived actin-rich filopodial processes that bridge these cells. He made important points that indicated that because virus particles are free to move in any direction, changes in receptor-envelope affinity dictated the cumulative unidirectional flow of particles along cytonemes towards the cell body of uninfected cells. Virus movement along filopodia was shown to be dependent on an intact actin-myosin machinery as previously described [2]. Thus MLV and other retroviruses surf along these processes to regions of the cell that are vulnerable to viral entry, likely to regions where there is active cytoskeletal remodelling. These results reveal another example of viruses hijacking host machineries to allow for efficient spreading of the infection from cell to cell.

Another important mediator of viral entry was highlighted by **Michel Tremblay **whose work has historically focused on integral membrane-spanning intracellular adhesion molecules (ICAMs) that are incorporated within the envelopes of retroviruses. Focusing on ICAM-1, a known host factor that dramatically enhances infectivity of virions [3], Michel Tremblay demonstrated that ICAM-1 interacts with its receptor, LFA-1 in microdomains and clusters at the cell surface of primary cells. This was shown to favor the release of viral capsids into target cells rather than endocytosis of virions. Similar to receptor-ligand interactions, the lateral diffusion of LFA-1 and its subsequent clustering were shown to be necessary to confer infectivity. Thus, the ICAM-1/LFA-1 ligand/receptor interaction facilitates infection, but is also important for the generation of the virological synapse during cell-to-cell transmission of retroviruses (described below) and thus represents a critical early step in infection.

**Boashan Zhang **(R. Montelaro lab) identified the host receptor for the ungulate equine infectious anemia virus (EIAV), a lentivirus that infects cells of the monocyte-macrophage lineage to cause progressive degenerative diseases without clinical immunodeficiency. This was identified as equine lentivirus receptor-1 (ELR1) that is related to the family of TNF receptor (TNFR) proteins [4]. With the aim of dissecting the molecular mechanisms of viral entry, this group described studies in which it mapped the domain of ELR1 that interacts with the EIAV Env to an amino-terminal cysteine-rich domain. It is clear that this work will provide a deeper understanding of some of the first virus-host interactions required for entry of this retrovirus.

The transmission of HIV-1 from dendritic cells to CD4^+ ^T cells represents one of the crucial stages for the establishment of infection [5]. **Li Wu **provided some insight into host factors that influence cell-to-cell transmission from dendritic cells to T cells. He presented some intriguing findings on how host gene (CD4 and DC-SIGN) expression levels influence HIV-1 infection and subsequent transmission from dendritic cells to T cells. This group provided evidence that CD4 expression levels could dramatically impact on viral transmission. Co-expression of CD4 strongly inhibited DC-SIGN-mediated HIV-1 transmission to T cells, and this was also echoed in studies in which Nef [6] was expressed in dendritic cells to downregulate CD4 levels. Furthermore, DC-SIGN expression levels were conversely upregulated by Nef and this impacted positively on HIV-1 transmission to T cells. Cumulatively, the results indicate that dendritic cells not only mediate HIV-1 *trans*-infection, but to facilitate cell-to-cell transmission, they can also be productively infected in order to express Nef at a later stage.

The final talk of this session was from **Quentin Sattentau **who extended his work on deciphering the mechanisms involved in the cell-to-cell transfer of HIV-1 between T cells. His group was instrumental in demonstrating that this occurs via the formation of a virological synapse that allows for efficient infection of neighboring cells for HIV-1 [7], a phenomenon that is also observed during HTLV-1 dissemination [8]. It has been appreciated for several years that cell-to-cell transmission relies on critical events that require a functional host cell cytoskeleton and clustering (or polarization) of cell surface receptors such as CD4 and cytoskeletal components. In this work, Sattentau evaluated the contributions of the cellular trafficking machinery (vesicles and cytoskeleton) and identified a vesicular compartment that could contribute to cell-to-cell transmission. The involvement of the microtubule-based cytoskeleton was also shown to be involved not only because the microtubule organizing center repositions proximally to the virological synapse, but also because the depolymerization of microtubules leads to the disruption of Gag and Env polarization at the synapse. Gag and Env were found in a spontaneously-formed, tetraspanin-rich vesicular compartment containing CD63, CD81 and CD9 at the plasma membrane in HIV-1-infected primary T cells. Sattentau suggested that this vesicular compartment shares some similarity to that found in the T cell secretory apparatus and thus would be enabling for HIV-1 transmission by targeting viral components to the virological synapse and promoting viral transmission and dissemination.

## Post-entry restrictions to retroviral infection

The recent discovery that host proteins TRIM5α and APOBEC3G are able to potently restrict retroviral infection, and that retroviruses have evolved mechanisms to counter these restrictions [9,10], has led to a tremendous increase in the number of studies aimed at understanding the early post-entry phase of retroviral infection. The session on post-entry restrictions began with a talk from **Paul Bieniasz **in which he described experiments designed to investigate whether diverse TRIM family members could inhibit HIV-1 infection if artificially targeted to the incoming capsid. Indeed, a number of TRIMs could disrupt HIV-1 infectivity when fused to cyclophilin A, which binds capsid. Gag chimeras in which the HIV-1 capsid domain was replaced with that of SIV (which lacks the ability to bind cyclophilin A) escaped this restriction. Some of these findings were echoed in a talk and a recent paper [11] from **Melvyn Yap **(J. Stoye lab) who presented data indicating that fusing heterologous coiled-coil domains to sequences that target capsid (e.g., cyclophilin A) can restrict HIV-1 infection. In this case, specificity was demonstrated by the ability of mutations in capsid that block cyclophilin A binding to reverse the restriction. From these studies emerged the concept that the presence of two independent domains, one that provides coiled-coil or multimerization function and the other that possesses Gag targeting activity, is in some cases sufficient to generate a restriction factor. In his talk, P. Bieniasz described a study that has taken advantage of our increased knowledge of post-entry restriction factors to engineer HIV-1 variants that are able to bypass blocks imposed on HIV-1 infection by non-human primate cells. These HIV-1 variants display greatly enhanced ability to replicate in rhesus macaque cells, making them potentially useful in developing non-human primate models for HIV-1 infection.

The mechanism of TRIM5α-mediated post-entry restriction was explored in a presentation by **Edward Campbell **from T. Hope's laboratory. While it has been demonstrated that proteasome inhibitors are not able to relieve the block to HIV-1 infection imposed by rhesus TRIM5α [12], Campbell and coworkers nevertheless observed that proteasome inhibition could rescue the defect in post-entry synthesis of viral DNA but could not reverse the block in the synthesis of 2-LTR circles. These results imply that TRIM5α imposes a block at more than one step in the post-entry pathway. Visualization of incoming virus (using GFP-Vpr) in cells expressing rhesus TRIM5α and treated with proteasome inhibitors revealed the accumulation of viral particles in rhesus TRIM5α cytoplasmic bodies. These observations suggest that TRIM5α may sequester incoming viral cores and induce their proteasome-mediated degradation [13].

Several presentations on post-entry restriction imposed by APOBEC3G and related APOBEC proteins began with an unexpected twist from **Klaus Strebel's **lab. H. Takeuchi and coworkers observed that replication of SIVagm in human T-cell lines requires Vif, but that no deamination is evident in Vif's absence. Further investigation into this phenomenon indicated that cyclophilin A is incorporated into Vif-deficient SIVagm virions but is absent from WT SIVagm virions [as reported previously [14,15]]. The relevance of cyclophilin A incorporation in the Vif(-) replication defect was demonstrated by the finding that replication of SIVagm in cyclophilin A knock-out cells or in cells treated with cyclosporine A (which blocks cyclophilin A activity) is Vif-independent. These results reveal a novel APOBEC-independent role for Vif in promoting SIV infection.

**Mariana Marin **(D. Kabat's lab) presented work aimed at identifying factors associated with APOBEC3G using mass spectrometry analyses. Over 100 proteins, most with mRNA-binding activity, were identified [16]. Their association with APOBEC3G was mRNA-specific, as binding was released by RNaseA treatment. The authors also observed that APOBEC3G is part of a polysomal population and could bind many diverse mRNAs, including viral genomic RNA. These results suggested that APOBEC3G complexes might be involved in regulating retroviral gene expression patterns at the level of mRNA export, translation and stability.

Initial studies strongly suggested that the major mechanism by which APOBEC3G blocks HIV-1 infectivity is through deamination of the nascent viral DNA post-entry, and consequent G-to-A hypermutation of the viral genome. However, several lines of evidence have suggested recently that APOBECs can restrict retroviruses and other viruses (e.g., hepatitis B virus) through deamination-independent mechanisms [17]. **Reubin Harris **addressed this controversial issue in his presentation. He observed that APOBEC3B and APOBEC3F are able to inhibit the retrotransposition of human long interspersed element 1 (L1). Significantly, catalytically inactive mutants of APOBEC3B are still able to inhibit L1 retrotransposition [18]. In contrast, deamination activity of human APOBEC3G was required for full levels of anti-HIV activity in this study. Harris also investigated the behavior of the APOBECs from artiodactyls (e.g., cattle, sheep, and pigs) and found a human-APOBEC3F-like protein that displays anti-HIV-1 activity that was not counteracted by HIV-1 Vif. Again, catalytic activity was a major part of the mechanism. Thus, depending on the retroid target, DNA cytosine deamination may or may not be an integral part of the restriction mechanism.

In the final talk of this session, **Vineet KewalRamani **presented work from his lab on a novel post-entry inhibitor of lentiviral infection, a truncated SR-family protein. This factor inhibits infectivity of primate lentiviruses (but not that of MLV) early post-entry by disrupting the stability and/or trafficking of the incoming viral genome. Analysis of HIV-1/MLV chimeras identified CA as the viral determinant of sensitivity to the antiviral factor. Indeed, the KewalRamani lab was able to select for HIV-1 variants that escaped this restriction and demonstrate that the changes responsible for escape mapped to CA. This antiviral factor thus provides an important new tool for understanding early post-entry steps in the HIV-1 replication cycle.

## Nuclear import and integration

The third session of the conference began with a presentation from **Frederic Bushman **focused on the targeting of retroviral integration. Previous studies from this group found that transcription units are favored for HIV-1 integration [19,20]; in contrast, MLV prefers to integrate at transcription start sites [21] whereas avian sarcoma-leukosis virus (ASLV) integration sites are nearly random [19,22]. The integrase (IN) enzyme itself appears to be the major viral determinant of target site selection [23], and the host factor lens epithelium-derived growth factor/p75 (LEDGF/p75) is an important player in this process [24]. To gain more information about HIV-1 target site selection, Bushman's lab used the powerful "454" sequencing method [25] to obtain 40,000 new sites of HIV-1 integration in infected Jurkat T cells. In addition to confirming the preference of HIV-1 integration for transcriptionally active regions, this study also showed that a collection of histone post-translational modifications positively associated with transcription had a stimulatory effect on integration whereas DNA methylation had a negative effect.

**Stuart Le Grice **provided a progress report on his laboratory's efforts, in collaboration with NCI-Frederick's Molecular Targets Discovery Program, NICHD in Bethesda, and the University of Pittsburgh, to develop selective inhibitors of HIV-1 ribonuclease H (RNase H). A total of ~250,000 compounds have been screened in this project, and several potent and specific inhibitors of HIV-1 RNase H have been identified. One of these, β-thujaplicinol, has also been shown to synergize with a nonnucleoside RT inhibitor, indicating that both the DNA polymerase and RNase H active sites can be simultaneously targeted. Structural studies aimed at defining the binding sites for these inhibitors are underway.

Returning to the role of LEDGF/p75 in HIV-1 integration, **Eric Poeschla **presented the results of his rather heroic efforts to intensify the knock-down of LEDGF/p75 using stable hairpin RNAs (shRNAs) expressed from lentiviral vectors. Previous reports had indicated that LEDGF/p75 binds HIV-1 IN, promotes IN nuclear localization and prevents its degradation by tethering the protein to chromatin (e.g., [26]). However, discordant results had been obtained regarding the impact of LEDGF/p75 depletion on HIV-1 infectivity. By intensifying the knock-down strategy, Poeschla's lab was able to virtually eliminate LEDGF/p75 expression, and, in particular, to strip detectable LEDGF/p75 from the DNase- and salt-releasable chromatin fraction. As a consequence, HIV-1 infectivity was reduced by ~30-fold. Feline immunodeficiency virus (FIV) infectivity was also greatly reduced; in contrast, MLV, whose IN protein does not bind LEDGF/p75, was unaffected. Rescue of HIV-1 infectivity was restored by adding back siRNA-resistant LEDGF/p75. Overexpression of the IN-binding domain of LEDGF/p75 also potently blocked HIV-1 infectivity [27]. The authors hypothesized that the failure to observe major defects in HIV-1 infectivity in previous LEDGF/p75 siRNA experiments was due to the presence of a small but functionally significant pool of chromatin-bound LEDGF/p75 that resisted depletion.

Continuing with the LEDGF/p75 theme, **Alan Engelman **reported their use of an alternative strategy to eliminate LEDGF/p75 expression; namely, the creation of mouse LEDGF/p75 knock-out cells. Infection of these cells with HIV-1 vectors was markedly reduced, whereas MLV infectivity was unaffected. Interestingly, preintegration complexes (PICs) isolated from LEDGF/p75 knock-out cells were defective for integration in vitro and this defect was shown to be due to the lack of LEDGF/p75 in the PICs. These findings suggest that LEDGF/p75 is an essential component of the HIV-1 PIC.

Post-translational modifications of viral proteins are now becoming important for their activities during virus replication in LTR transactivation, for instance. For HIV-1 IN, this was also shown to be the case by **Lara Manganaro **(M. Giacca lab) who demonstrated that p300, a cellular acetyltransferase that regulates chromatin conformation through the acetylation of histones, also acetylates IN and controls its activity [28]. Acetylation of C-terminal lysines (Lys264, 266 and 273) and conserved (in retroviruses) regions of IN were shown to be important for DNA association, IN strand transfer activity and possibly IN protein stability in HIV-1 infected cells. Future work will focus on temporal nature of acetylation and what other functions of IN are affected by this post-translational modification.

## Gene expression/regulation of retroviral gag and RNA

**Kathy Boris-Lawrie **opened this session and spoke about virus-host interactions involving the activities of the post-transcriptional control element (PCE). The PCE is a highly structured RNA element in the 5'untranslated region of RNA that was identified in some retroviruses including avian spleen necrosis virus and Mason-Pfizer monkey virus [29,30], reticuloendotheliosis virus strain A and HTLV-1 (Bolinger and Boris-Lawrie, unpublished). Affinity chromatography using the PCE as a bait followed by mass spectrometry analyses identified the RNA helicase A (RHA), DDX9 in the eluate, a protein with well-described roles in transcription, and less well established roles in retroviral splicing and nuclear export [31]. The specificity of RHA interaction was shown by co-immunoprecipitation of Flag-tagged RHA and PCE and lack of co-immunoprecipitation with a bank of nonfunctional PCE mutants. The rescue of co-immunoprecipitation by compensatory mutations that restore the stem-loop structure of the PCE determined that RHA specifically recognized the double stranded structure of PCE. Downmodulation of this RNA helicase by siRNA together with polysome analysis revealed that it is necessary for Gag expression whereby downmodulation did not affect RNA splicing, export or steady state levels, but reduced polysome association of the gag mRNA. By contrast, translation of global cellular mRNA was unaffected [32]. This work revealed that RHA might be a general factor that specifically associates with selected structured RNAs of both viral and human origin to facilitate their translation.

**Karen Beemon **described her group's recent efforts to understand the regulation of Rous sarcoma virus (RSV) post-transcriptional regulation that relies on peculiarities of the RSV RNA sequences. Normally, an RNA undergoes degradation via nonsense-mediated decay (NMD) when a premature termination (or stop) codon (PTC) is encountered by ribosomes in an mRNA and this in many cases is dependent on the deposition of a multi-protein complex (the exon junction complex or EJC) on the RNA and splicing events in the nucleus [33]. In earlier work from Beemon's lab, RSV *gag *RNA was shown to be a substrate for NMD when PTCs were introduced in *gag *RNA [34]. However, RSV genomic RNAs as well as other retroviral RNAs are not only considered aberrant because of the lack of splicing events, the presence of introns and the possible absence of EJCs, but also appear to be canonical substrates for NMD with their unusually long 3'UTRs (that for *gag *RNA is >7 kb) [35]. The Beemon lab uncovered a 401-nucleotide cis-acting sequence downstream of the *gag *termination codon that prevented recognition of the *gag *mRNA, and more specifically, the *bona fide *termination codon by the NMD machinery [36]. While it remains to be determined whether similar regulatory sequences are present in other retroviruses to prevent recognition of this cellular RNA surveillance machinery, these results point to yet another complex regulatory circuit to maintain levels of retroviral RNAs and to ensure their utilization for structural protein synthesis.

A little further upstream in the gene expression phase of HIV-1 replication are splicing regulation and 3'end processing of RNA. These processes are highly complex for retroviruses like HIV-1 due to the presence of multiple positive and negative regulatory elements in the viral genome and the activities of multiple host proteins in controlling their activity [37]. **Alan Cochrane **demonstrated that the modulation of host protein gene [Ser- and Arg-rich (SR) splicing factors, hnRNPs] expression impacted on splicing and 3'end-processing of HIV-1 RNAs and modulated expression of HIV-1 structural proteins. These studies again highlight the complex regulation that occurs during the maturation of retroviral RNAs. The key role of polyadenylation in expression of viral genes was exploited by this group to develop a strategy to selectively inhibit HIV-1 expression by targeting the binding of modified U1 snRNPs to regions of RNA adjacent to the polyadenylation signal. The magnitude of the inhibition observed coupled with the high degree of conservation of the sequences targeted suggests that this approach might have an application in targeting HIV-1 in a gene therapy approach.

In the first of several talks focused on retroviral Gag targeting, **Akira Ono **presented work from his lab on the viral and cellular determinants of HIV-1 Gag targeting to the plasma membrane and multivesicular body (MVB). Building on previous results that the phosphoinositide phosphatidyl (4,5)bisphosphate [PI(4,5)P_2_] plays an important role in Gag targeting [38], Ono examined the binding of HIV-1 Gag to liposomes either containing or lacking PI(4,5)P_2_. These studies indicated that the presence of PI(4,5)P_2 _enhanced binding of WT HIV-1 Gag but not that of Gag mutants containing mutations in a basic region of matrix (MA) implicated in Gag targeting. These results are consistent with the recent structural demonstration of a direct interaction between HIV-1 Gag and PI(4,5)P_2 _[39]. To investigate the viral determinants of Gag targeting, Ono examined the localization of various HIV-1 Gag mutants and chimeras, both in the presence or absence of PI(4,5)P_2 _depletion. Intriguingly, localization of Gag to the MVB (either upon PI(4,5)P_2 _depletion or mutation of the MA basic domain) was prevented by mutation or removal of the NC domain. These results implicate NC in the targeting of Gag to the MVB, perhaps by promoting its assembly-induced retention at the MVB.

While Gag is a powerful targeting protein that can alone direct virus-like particle formation from cells, Gag also encounters multiple host proteins and machineries during its transit in the cell. Gag is also considered critical to genomic RNA trafficking, selection and encapsidation. Much attention has been paid to the multitude of host proteins involved in transporting HIV-1 RNAs out of the nucleus[40] since this process still represents a suitable therapeutic target. Once the RNA gets into the cytoplasm however, few details are known about the fate of retroviral RNAs. While the genomic RNA must be both translated and encapsidated, trafficking may rely in part on vesicular [41,42] and another part on RNP trafficking mechanisms [43]. **Andrew Mouland **showed work demonstrating the involvement of hnRNP A2 in nucleocytoplasmic and intracytoplasmic trafficking of the genomic RNA using siRNA-mediated knockdown and in situ fluorescence imaging techniques. This work identified the microtubule organizing center as a site via which HIV-1 genomic RNA enters the cytoplasm [44], a site that has been shown to be targeted by incoming viral capsids before entry into the nucleus for HIV-1 and other viruses [45]. This work highlighted the importance of host proteins and machineries in the targeting of viral components during the gene expression and assembly phases of the retroviral replication cycle.

**Amanda Dalton **from V. Vogt's lab, in collaboration with D. Murray's group, described studies aimed at measuring the association of HIV-1 MA with liposomes of varying composition. These studies had their genesis in similar work from this group on RSV MA. The MA used in the binding assays was either myristylated or not, and was either monomeric or artificially dimerized. The results emphasized the importance of negatively charged lipids in MA binding to membrane. Furthermore, dimerization greatly increased the affinity of HIV-1 MA for liposomes. This and previous work from these authors emphasizes the role of electrostatic interactions and Gag multimerization in the binding of both HIV-1 and RSV Gag to membrane.

In studies focused on defining the assembly pathway of HIV-1 Gag, **Delphine Muriaux **used subcellular fractionation techniques to examine the localization of Gag in transfected 293T cells and chronically infected MOLT T-cells. Cell lysates prepared from virus-expressing cells were fractionated on iodixanol gradients. The sedimentation of Gag was compared to that of markers for plasma membrane, late endosomes, small vesicles, and soluble proteins [46]. The results indicated that Gag was found in fractions that corresponded to both the plasma membrane and late endosomes. Genomic RNA was observed primarily in late endosomal and soluble fractions. Similar results were obtained in the MOLT T cells. These observations, which were confirmed by immunofluorescence and electron microscopy, led the authors to conclude that HIV-1 assembly in 293T and chronically infected T cells takes place at both the plasma membrane and in MVBs. Removal of the zinc fingers in the NC domain resulted in a shift in Gag localization from endosomal to recycling vesicle fractions, and a loss in the cosedimentation of Gag and genomic RNA.

## Assembly and release of retroviruses

Retroviral particle budding is promoted by small motifs in Gag known as late domains (for review, see [47-49]). These motifs stimulate virus release by interacting with components of the cellular endosomal sorting machinery, which regulate the delivery of cargo proteins into the MVB pathway, and the biogenesis of the vesicles that bud into MVBs. Three types of retroviral late domains have been characterized: Pro-Thr/Ser-Ala-Pro [P(T/S)AP], Pro-Pro-x-Pro (PPxY), and Tyr-Pro-x_n_-Leu (YPx_n_L). P(T/S)AP, the dominant HIV-1 late domain found in Gag-p6, binds Tsg101, a component of the ESCRT-I complex (endosomal sorting complex required for transport). PPxY late domains interact with ubiquitin ligases in the Nedd4 family, and YPxL motifs associate with Alix (also known as AIP1). Interestingly, although P(T/S)AP is the major late domain of HIV-1, p6 also bears a YPx_n_L-type motif that has been shown to bind Alix.

In his keynote address, **Wesley Sundquist **discussed several aspects of the cell biology and biochemistry of HIV-1 budding. He first described some of the cellular apparatus that associates with Tsg101. In addition to the ESCRT-1 components Vps28 and Vps37, Tsg101 also binds Hrs, Alix, the GGA proteins, and TOM1L1. Interestingly, TOM1L1 also interacts with Nedd4-like E3 ubiquitin ligases, raising the possibility that it might play a role in the recruitment of PPxY-containing retroviruses into the MVB pathway. Ubiquitination of cargo proteins is often (but not always) required for their sorting into MVBs, and there are several lines of evidence suggesting that ubiquitination of Gag itself may play a positive role in virus release. A number of components of the MVB machinery, including Hrs, Tsg101, and the ESCRT-II component EAP45, contain motifs that directly bind ubiquitin. HIV-1 Gag, for example, could interact with Tsg101 not only through its P(T/S)AP motif in p6 but also through ubiquitin moieties attached to several domains of Gag [50].

Purification and analysis of ESCRT-I complexes in Sundquist's lab revealed a heretofore unrecognized fourth component of ESCRT-I, referred to as EI4A (and variant EI4B).

Finally, Sundquist described his lab's studies on Alix. While it is now fairly clear that Alix is the major late-domain-interacting protein for EIAV, as mentioned above HIV-1 p6 also interacts with Alix. A role for Alix in HIV-1 release is most apparent when the Gag/Tsg101 interaction has been abolished. Structural studies with the central, Gag-binding domain of Alix revealed a V-shaped fold, with the YPx_n_L binding site lying inside the base of the V.

The continuing discovery of additional components of ESCRT and associated machinery adds to the complexity of the endosomal sorting (and virus budding) machinery. Sundquist pointed out that ~100 proteins are involved in endocytosis and that a comparable number of proteins may ultimately be implicated in MVB biogenesis. It will be of great interest to define which of this multitude of cellular factors are required for the release of HIV-1 and other retroviruses.

Previous studies from the lab of **Jaisri Lingappa **demonstrated that HIV-1 assembly proceeds through the formation of a series of discrete intermediates of 10S, 80S, 150S, and 500S, culminating in a 750S immature VLP [51]. The subcellular localization of these assembly intermediates was investigated by Lingappa and coworkers using membrane flotation techniques. The 10S complex was found to be cytosolic, the 80S/150S was in both cytosolic and membrane-associated fractions, whereas the 500S and 750S complexes were predominantly found in membrane. The assembly cofactor ABCE1 (formerly referred to as HP68 [52]) was present in both cytosolic and membrane fractions. Interestingly, in murine cells, which according to some studies display a defect in HIV-1 particle production [53,54], assembly is arrested at the stage of 80S/150S complex formation.

Several labs, including **Mark Marsh's**, have previously reported that in monocyte-derived macrophages HIV-1 assembly takes place primarily in a late endosome or MVB compartment (for review, see [55]). Mark Marsh expanded on this theme in his presentation and provided a more refined view of the compartment in which assembly occurs in this cell type. Using a combination of confocal microscopy and immuno-EM, the colocalization of Gag with a variety of tetraspannin markers previously used to define the late endosome (e.g., CD9, CD53, CD63, and CD81) was examined. Only partial overlap was observed between Gag and CD63 (as previously reported [56]), whereas colocalization of Gag with CD9 and CD81 was more extensive. Interestingly, organelles positive for CD9, CD53 and CD81 displayed a complex morphology with extensive internal membranes, suggesting that this compartment may be distinct from that in which CD63 is concentrated. A partial shift in CD63 localization was observed in HIV-infected cells, raising the possibility that HIV may alter the CD9-, CD53-, and CD81-containing compartment in infected macrophages.

An alternative perspective on the localization of HIV-1 assembly was provided by **Nolwenn Jouvenet **(P. Bieniasz lab). Jouvenet presented a series of results that were used to argue that HIV-1 assembly takes place on the plasma membrane irrespective of the cell type in which Gag is expressed. Chimeric Gag proteins that contain MVB-targeting signals were severely defective in virus release, whereas drugs that block late endosome mobility did not affect virus particle production, even in macrophages. These observations suggest that the localization of Gag to the MVB may be part of a non-productive assembly pathway. Thus, there currently exists a continuum of opinions in the field regarding the site of HIV-1 assembly: some have argued that MVB assembly predominates in all cell types, others believe that the plasma membrane is the major site of assembly regardless of cell type, and a third group of investigators has reported that the site of assembly is cell type-dependent, with HeLa and T-cells showing predominantly plasma membrane assembly and primary macrophages displaying a high level of MVB-associated assembly. Real-time imaging of infected cells will be helpful in resolving this debate.

**Paul Spearman **presented his lab's findings on the localization and function of the HIV-1 accessory protein Vpu, which possesses the ability to stimulate virus release from most human cell types. Some of these results were published recently [57]. Based on colocalization analyses with cellular markers, Vpu was observed to be concentrated in a recycling endosome compartment. Disruption of recycling endosome function with dominant-negative versions of Rab11a or myosin Vb blocked the ability of Vpu to promote virus release. Several reports have shown that the Env glycoprotein from the ROD_10 _strain of HIV-2 possesses a Vpu-like ability to enhance HIV-1 release; this activity of HIV-2 Env was also blocked by recycling endosome disruption. It has been postulated that Vpu acts by counteracting a cellular protein that delays virus release, thus favoring release over internalization of newly budded particles [57-59]. Given Vpu's localization in recycling endosomes and its limited presence on the plasma membrane, its ability to block the activity of a putatively surface-associated factor might be indirect rather than through a direct protein-protein interaction.

As mentioned above, HIV-1 budding is promoted by cellular machinery that normally functions in the biogenesis of vesicles that bud into late endosomes to form MVBs. This machinery includes the three multiprotein complexes ESCRT-I, II, and III. In human cells, the ESCRT-III complex is composed of a set of CHMP (for charged MVB) proteins [60]. **Heinrich Gottlinger's **lab has previously reported that overexpression of CHMP3 and CHMP4a proteins fused to red fluorescent protein (RFP) led to a potent dominant-negative inhibition of HIV-1 release [61]. At this meeting, Gottlinger reported the results of a study that examined the ability of CHMP3 overexpression to inhibit HIV-1 release. Because CHMP3 bears a highly basic N-terminal domain and a highly acidic C-terminal domain, Gottlinger postulated that an intramolecular interaction might occur that would lead to autoinhibition of CHMP3 function. According to this model, deletion of either N- or C-terminal domain would relieve the autoinhibition and activate the protein. Gottlinger presented evidence to support this model: N- and C-terminal domains were observed to interact, and removal of the C-terminal domain resulted in a protein capable of interfering with HIV-1 release. Activation of CHMP3 could also be induced by overexpression of a reported CHMP3-binding partner, the ubiquitin isopeptidase AMSH [62].

In the final talk of the conference, **Markus Thali **reported on the role of tetraspanins in virus release. Specifically, he presented data indicating that HIV-1 localizes to regions of the plasma membrane that are enriched in a set of tetraspanins that includes CD9, CD63, CD81, and CD82 [63]. The ESCRT-I components Tsg101 and Vps28 also concentrate in these microdomains, suggesting that these tetraspanin-enriched microdomains (TEMs) serve as platforms for virus budding. Providing functional data in support of this hypothesis, Thali showed that an antibody against CD9 inhibited HIV-1 release (consistent with an earlier report in which FIV release was inhibited by a different anti-CD9 antibody [64]), apparently by clustering TEMs. Interestingly, the budding of influenza virus was not inhibited by the anti-CD9 antibody, suggesting that orthomyxoviruses bud from plasma membrane microdomains distinct from those used by HIV-1.

**Figure 1:**
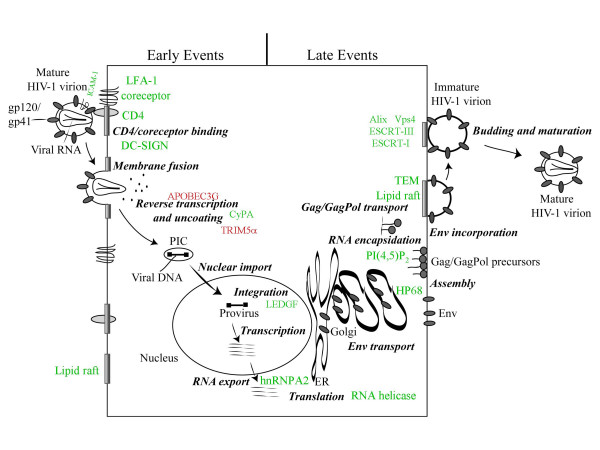
Cartoon of the HIV-1 replication cycle, with cellular factors that promote virus replication shown in green, and inhibitory factors in red. Details are provided in the text.

An overview of the HIV-1 replication cycle, with positive and negative host factors indicated, is illustrated in Figure 1.

## Authors' contributions

Both authors contributed equally to the inception and writing of the manuscript.
